# Supplementary material for: HSP90 Inhibitor, 17-DMAG, Alone and in Combination with Lapatinib Attenuates Acquired Lapatinib-Resistance in ER-positive, HER2-Overexpressing Breast Cancer Cell Line
Source: Cancers (Basel). 2020 Sep 15;12(9):2630. doi: 10.3390/cancers12092630 (PMC7564044; doi:10.3390/cancers12092630)
Supplement: Supplementary file 1 [file cancers-12-02630-s001.pdf]

cancers

MDPI

Article

HSP90 Inhibitor, 17-DMAG, Alone and in Combination with Lapatinib Attenuates Acquired Lapatinib-Resistance in ER-positive, HER2-Overexpressing Breast Cancer Cell Line

Hye Jin Lee [1], Seungho Shin [2], Jinho Kang [1], Ki-Cheol Han [3], Yeul Hong Kim [1], Jeoung-Won Bae [2] and Kyong Hwa Park [1,*]

[1] Division of Oncology/Hematology, Department of Internal Medicine, Korea University College Medicine, Seoul 02841, Korea; lhj5727@korea.ac.kr (H.J.L.); chinhokang@naver.com (J.K.); yhk0215@korea.ac.kr (Y.H.K.)

[2] Departments of Breast and Endocrine surgery, Korea University College Medicine, Seoul 02841, Korea; sshhere@naver.com (S.S.); kujwbae@korea.ac.kr (J.-W.B.)

[3] Center for Theragnosis, Biomedical Research Institute, Korea Institute of Science and Technology, Seoul 02456, Korea; biolord@kist.re.kr

* Correspondence: khpark@korea.ac.kr; Tel.: +82-2-920-6841

Received: 7 August 2020; Accepted: 12 September 2020; Published: date

Supplementary materials

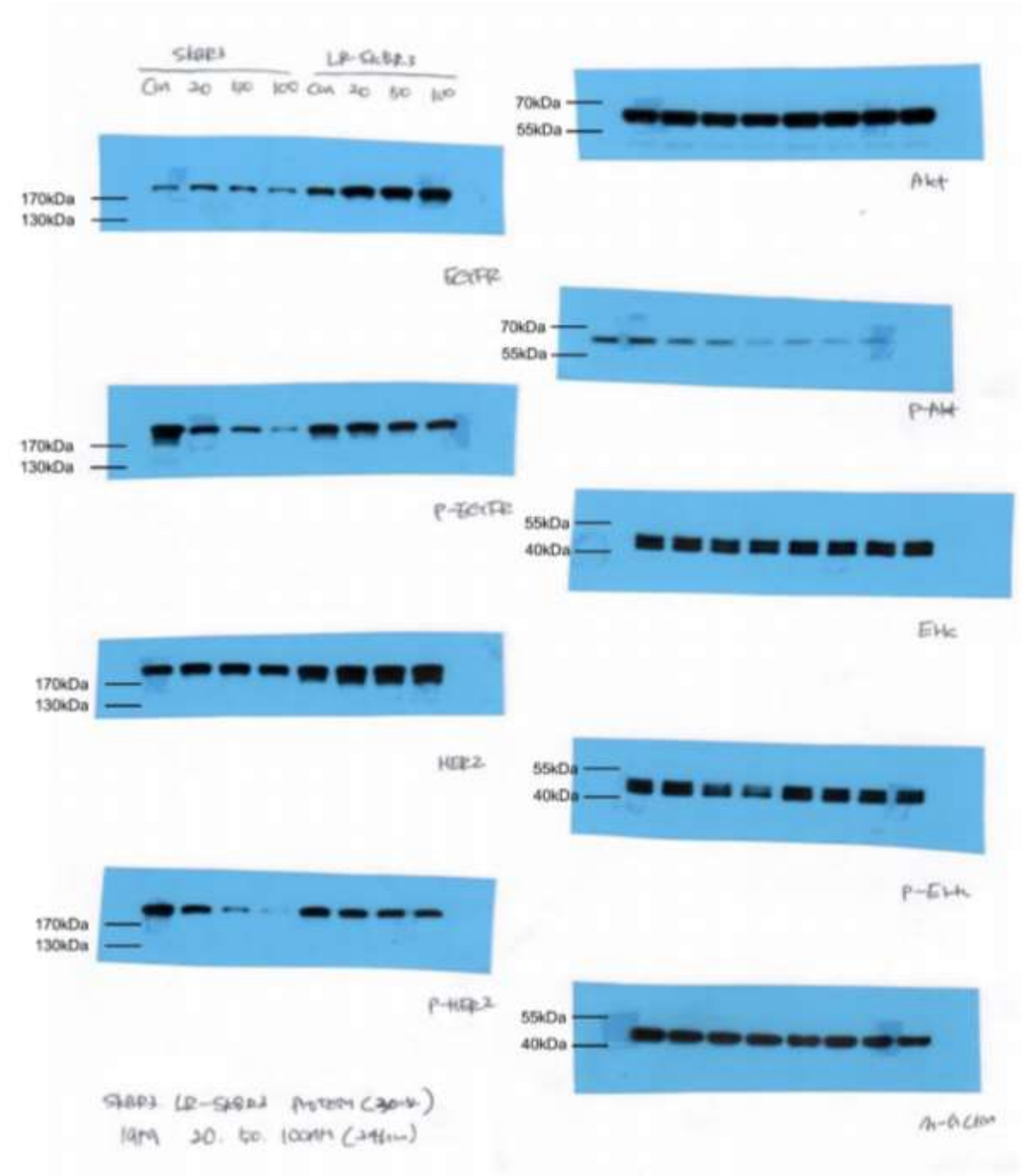

		EGFR	p-EGFR	HER2	p-HER2	Akt	p-Akt	ERK	p-ERK
Lane 1	**SKBR3-Con**	0.14	0.96	0.52	0.78	0.59	0.45	0.75	0.86
Lane 2	**SKBR3-Lapatinib 20nM**	0.30	0.63	0.86	1.28	0.97	0.74	1.22	1.41
Lane 3	**SKBR3-Lapatinib 50nM**	0.27	0.36	0.94	0.20	0.62	0.44	1.01	0.96
Lane 4	**SKBR3-Lapatinib 100nM**	0.22	0.15	1.05	0.02	0.86	0.49	1.29	0.93
Lane 5	**LR-SKBR3-Con**	0.52	0.75	1.08	0.69	0.81	0.16	0.99	1.16
Lane 6	**LR-SKBR3-Lapatinib 20nM**	1.16	0.79	1.37	0.57	0.72	0.13	0.81	0.87
Lane 7	**LR-SKBR3-Lapatinib 50nM**	1.18	0.68	1.52	0.55	0.83	0.17	0.99	0.98
Lane 8	**LR-SKBR3-Lapatinib 100nM**	1.15	0.52	1.44	0.53	0.67	0.16	0.91	0.83

Figure S1. Original image and band density analysis in Figure 1B for western blot in SKBR3 and LR-SKBR3 cell lines.

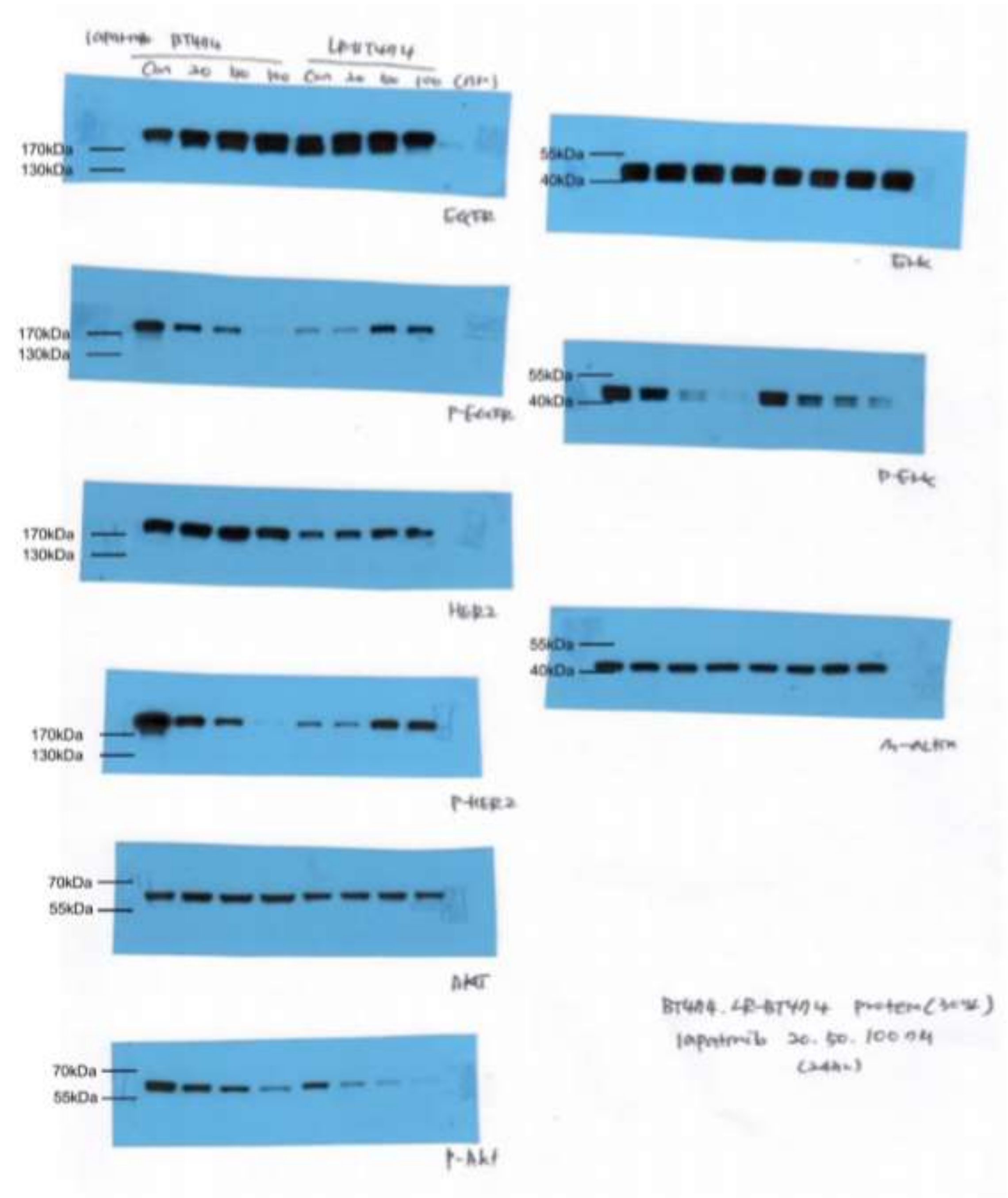

		EGFR	p-EGFR	HER2	p-HER2	Akt	p-Akt	ERK	p-ERK
Lane 1	BT474-Con	0.57	1.07	0.97	1.65	0.67	0.96	1.35	1.05
Lane 2	BT474-Lapatinib 20nM	1.01	0.64	1.36	0.85	1.02	0.83	1.55	0.92
Lane 3	BT474-Lapatinib 50nM	1.27	0.45	1.48	0.55	0.99	0.65	1.68	0.23
Lane 4	BT474-Lapatinib 100nM	1.28	0.01	0.92	0.00	0.85	0.20	1.49	0.12
Lane 5	LR-BT474-Con	1.42	0.20	0.51	0.36	0.79	0.64	1.44	1.20
Lane 6	LR-BT474-Lapatinib 20nM	1.54	0.12	0.60	0.20	0.76	0.23	1.40	0.68
Lane 7	LR-BT474-Lapatinib 50nM	1.31	0.55	0.57	0.59	0.66	0.09	1.32	0.48
Lane 8	LR-BT474-Lapatinib 100nM	0.98	0.47	0.48	0.55	0.63	0.02	1.19	0.25

Figure S2. Original image and band density analysis in Figure 1B for western blot in BT474 and LR-BT474 cell lines.

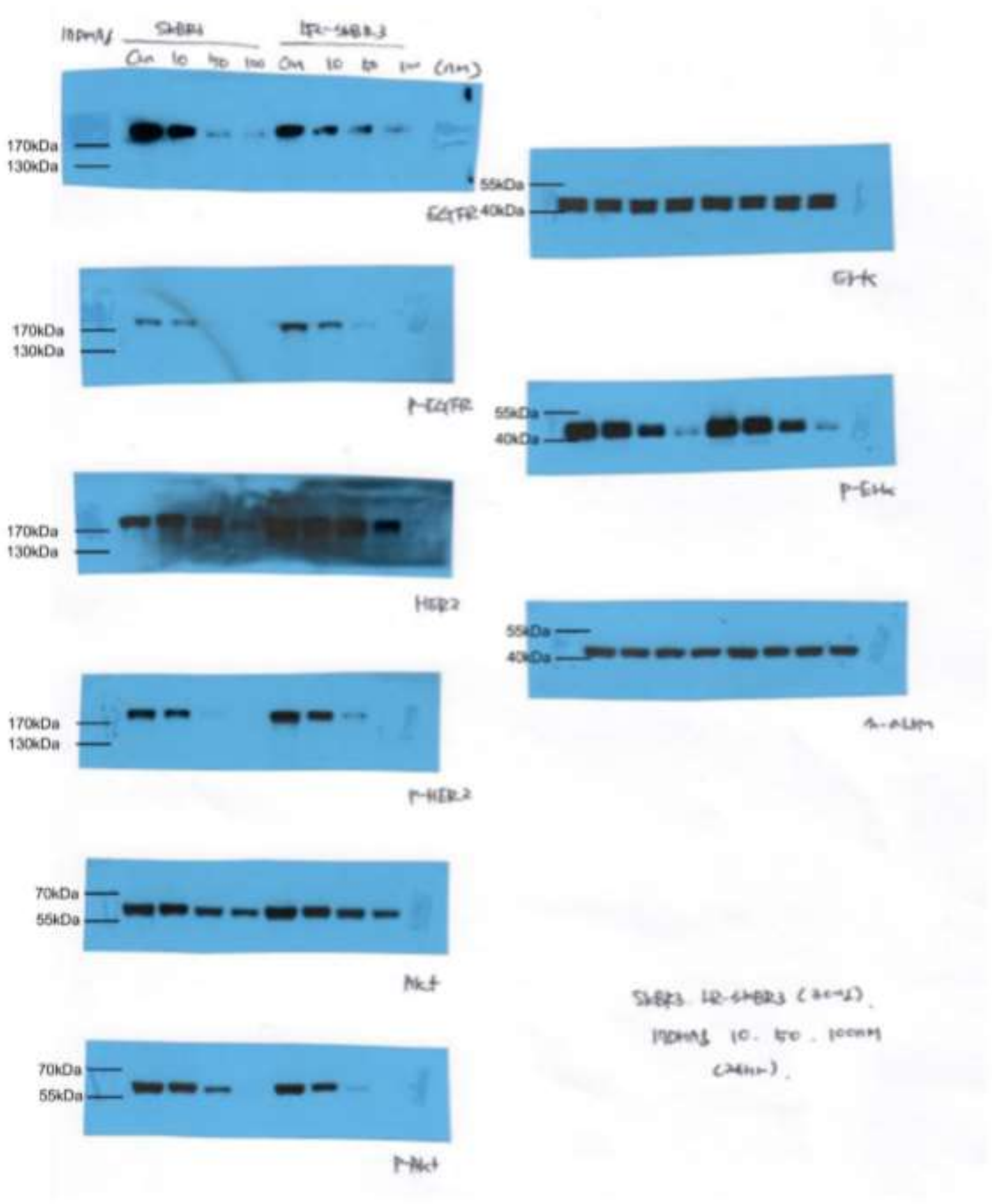

		EGFR	p-EGFR	HER2	p-HER2	Akt	p-Akt	ERK	p-ERK
Lane 1	**SKBR3-Con**	2.04	0.57	1.08	1.00	1.20	1.20	1.21	1.59
Lane 2	**SKBR3-17-DMAG 10nM**	1.42	0.49	2.34	0.82	1.25	1.14	1.15	1.55
Lane 3	**SKBR3-17-DMAG 50nM**	0.11	0.33	2.72	0.22	0.88	0.56	1.13	0.94
Lane 4	**SKBR3-17-DMAG 100nM**	0.05	0.22	2.40	0.18	0.75	0.17	1.34	0.31
Lane 5	**LR-SKBR3-Con**	0.90	0.67	2.77	1.27	1.28	1.00	1.08	1.58
Lane 6	**LR-SKBR3-17-DMAG 10nM**	0.47	0.49	2.95	1.07	1.22	0.81	1.16	1.67
Lane 7	**LR-SKBR3-17-DMAG 50nM**	0.32	0.13	3.14	0.28	0.94	0.12	1.25	0.93
Lane 8	**LR-SKBR3-17-DMAG 100nM**	0.12	0.01	1.97	0.01	0.54	0.02	1.14	0.15

Figure S3. Original image and band density analysis in Figure 4B for western blot in SKBR3 and LR-SKBR3 cell lines.

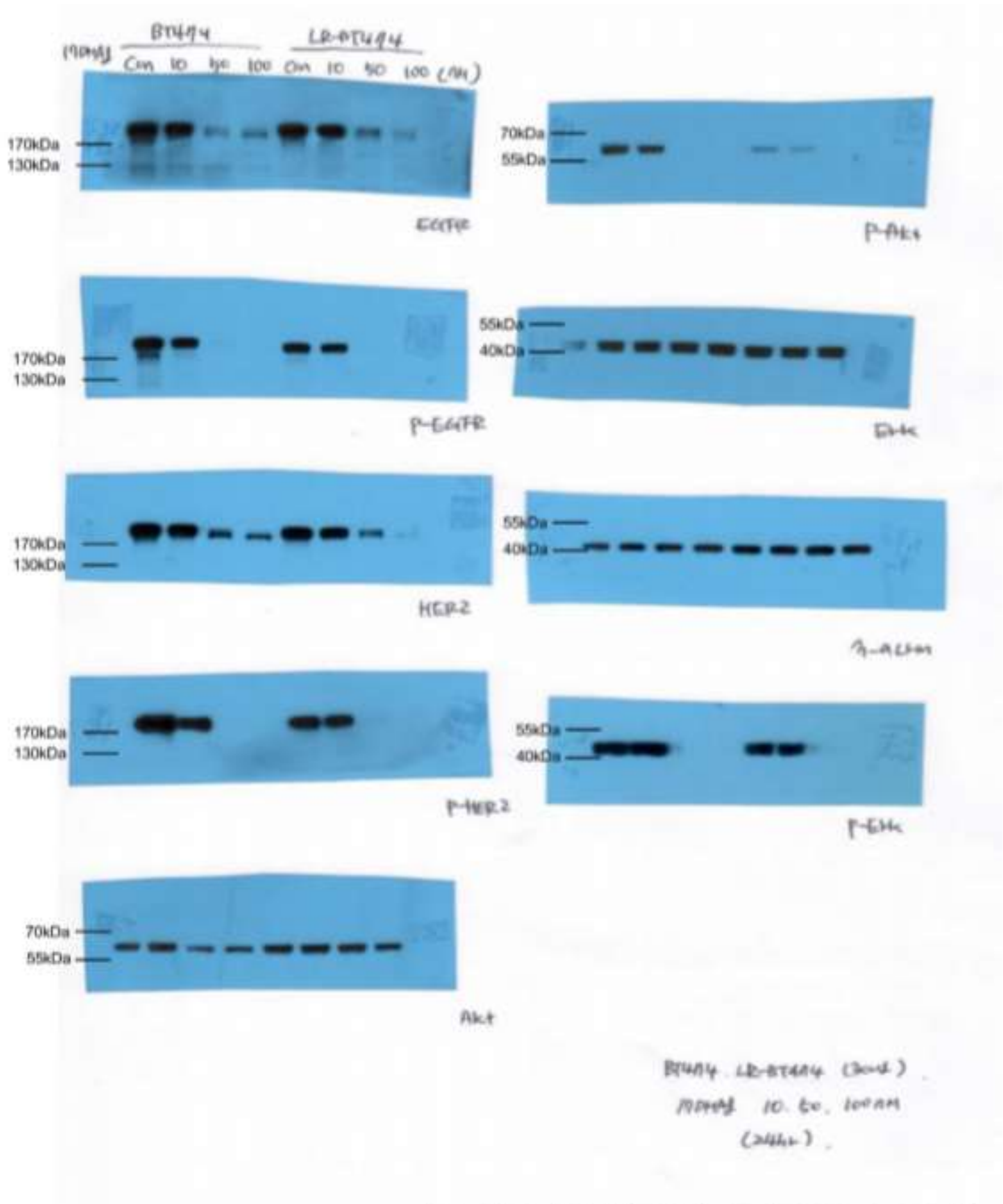

		EGFR	p-EGFR	HER2	p-HER2	Akt	p-Akt	ERK	p-ERK
Lane 1	**BT474-Con**	2.66	2.68	2.71	4.62	1.18	1.27	0.43	2.45
Lane 2	**BT474-17-DMAG 10nM**	1.74	1.29	1.84	1.88	1.36	0.88	1.05	2.47
Lane 3	**BT474-17-DMAG 50nM**	0.46	0.21	0.79	0.10	0.70	0.30	1.21	0.06
Lane 4	**BT474-17-DMAG 100nM**	0.43	0.16	0.52	0.03	0.76	0.16	1.20	0.05
Lane 5	**LR-BT474-Con**	1.21	0.88	1.44	1.65	0.84	0.24	0.89	1.16
Lane 6	**LR-BT474-17-DMAG 10nM**	1.21	0.79	1.09	1.52	1.00	0.23	1.06	1.21
Lane 7	**LR-BT474-17-DMAG 50nM**	0.46	0.07	0.36	0.28	0.87	0.07	0.96	0.13
Lane 8	**LR-BT474-17-DMAG 100nM**	0.26	0.03	0.02	0.20	0.73	0.01	0.75	0.09

Figure S4. Original image and band density analysis in Figure 4B for western blot in BT474 and LR-BT474 cell lines.

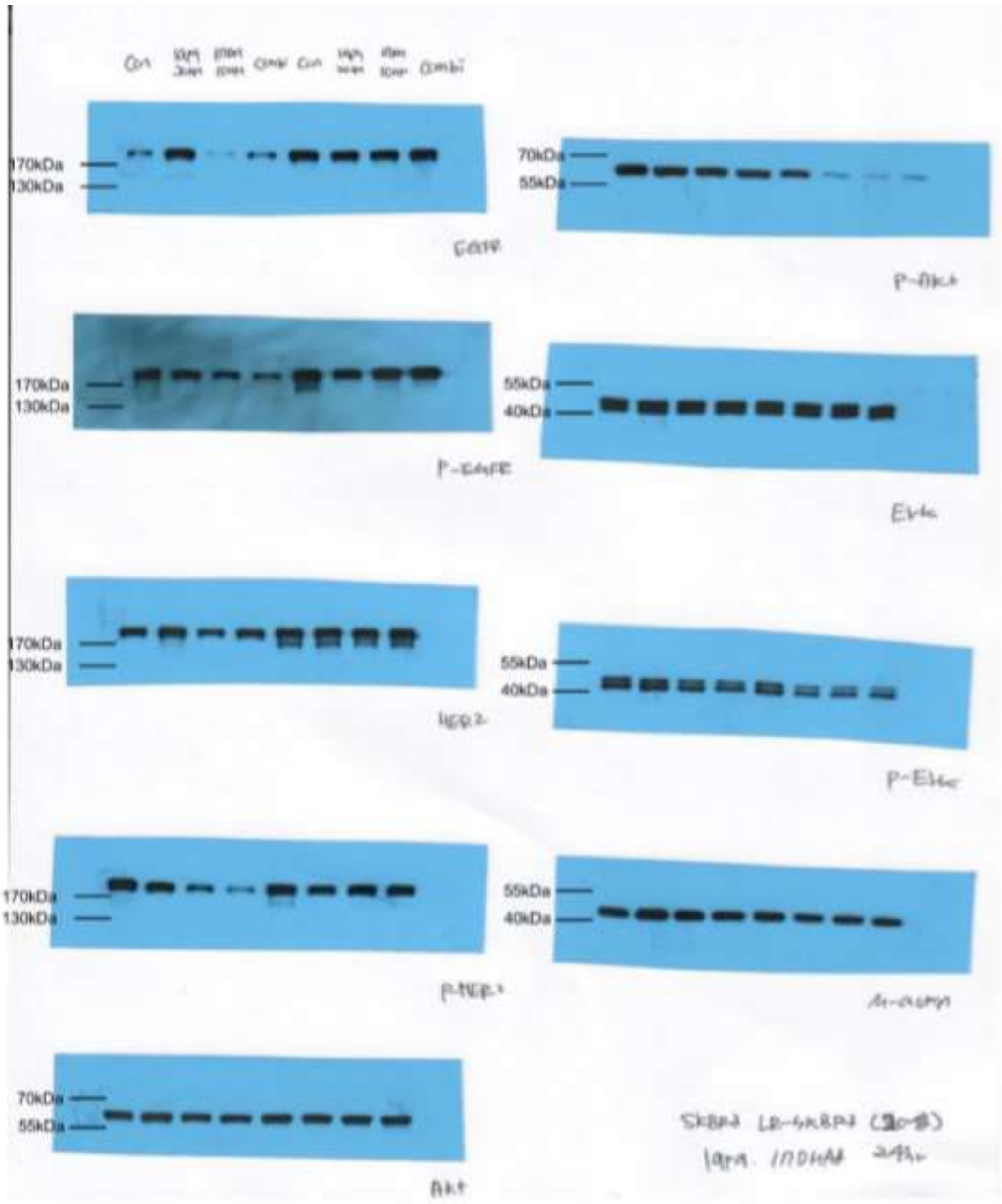

		EGFR	p-EGFR	HER2	p-HER2	Akt	p-Akt	ERK	p-ERK
Lane 1	**SKBR3-Con**	0.47	1.67	0.72	1.34	0.80	1.36	1.21	0.89
Lane 2	**SKBR3-Lapatinib 20nM**	0.94	1.03	0.67	0.66	0.58	0.79	0.89	0.74
Lane 3	**SKBR3-17-DMAG 10nM**	0.15	0.97	0.47	0.48	0.63	0.86	1.06	0.62
Lane 4	**SKBR3-Lapa 20nM+17DM 10nM**	0.46	0.81	0.69	0.27	0.69	0.87	1.15	0.55
Lane 5	**LR-SKBR3-Con**	1.28	1.54	1.13	1.16	0.76	0.64	1.08	0.83
Lane 6	**LR-SKBR3-Lapatinib 20nM**	1.56	1.03	1.51	0.86	0.97	0.17	1.39	0.57
Lane 7	**LR-SKBR3-17-DMAG 10nM**	1.38	1.09	1.32	0.97	1.00	0.06	1.44	0.56
Lane 8	**LR-SKBR3-Lapa 20nM+17DM 10nM**	1.62	1.25	1.36	0.93	0.98	0.11	1.38	0.71

Figure S5. Original image and band density analysis in Figure 5B for western blot in SKBR3 and LR-SKBR3 cell lines.

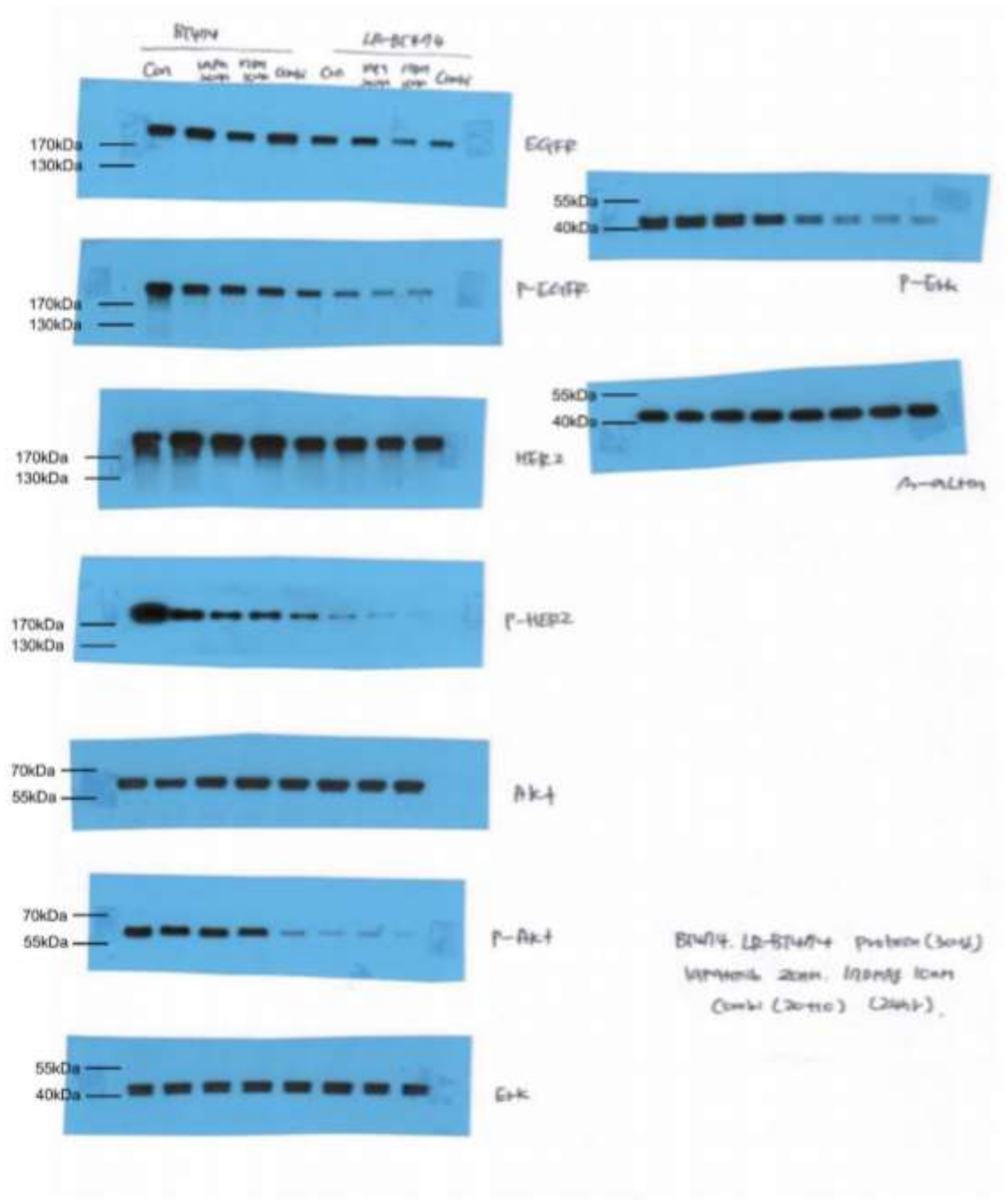

		EGFR	p-EGFR	HER2	p-HER2	Akt	p-Akt	ERK	p-ERK
Lane 1	**BT474-Con**	0.77	1.22	1.18	1.77	0.99	0.84	0.57	0.86
Lane 2	**BT474-Lapatinib 20nM**	0.86	0.88	1.63	0.83	0.93	0.82	0.57	0.85
Lane 3	**BT474-17-DMAG 10nM**	0.56	0.64	1.15	0.58	1.04	0.76	0.62	0.88
Lane 4	**BT474-Lapa 20nM+17DM 10nM**	0.72	0.56	1.34	0.52	1.20	0.60	0.59	0.54
Lane 5	**LR-BT474-Con**	0.47	0.46	1.02	0.47	1.02	0.19	0.65	0.31
Lane 6	**LR-BT474-Lapatinib 20nM**	0.45	0.25	0.79	0.25	1.02	0.15	0.76	0.20
Lane 7	**LR-BT474-17-DMAG 10nM**	0.29	0.23	0.93	0.17	1.18	0.21	0.75	0.21
Lane 8	**LR-BT474-Lapa 20nM+17DM 10nM**	0.27	0.16	0.97	0.03	1.20	0.05	0.78	0.18

Figure S6. Original image and band density analysis in Figure 5B for western blot in BT474 and LR-BT474 cell lines.
